# Inclusive Leadership and Employees’ Helping Behaviors: Role of Psychological Factors

**DOI:** 10.3389/fpsyg.2022.888094

**Published:** 2022-07-07

**Authors:** Samina Qasim, Muhammad Usman, Usman Ghani, Kalimullah Khan

**Affiliations:** ^1^Department of Business Administration, Iqra University, Karachi, Pakistan; ^2^School of Management, Xiamen University, Xiamen, China; ^3^College of Education, Zhejiang University, Hangzhou, China; ^4^Department of MBA, School of Graduate Studies, Kardan University, Kabul, Afghanistan

**Keywords:** inclusive leadership, helping behavior, employees psychological engagement, psychological safety, health sector

## Abstract

Based on social learning theory, the present study investigates the influence of inclusive leadership on employees’ helping behaviors. Further, psychological mechanisms (psychological safety and psychological engagement) are investigated in the relationship between inclusive leadership and employees’ helping behaviors. The data was collected in three time-lags through a questionnaire from 409 nurses working in the health sector of Pakistan. The collected data was analyzed through IBM-SPSS and AMOS to test the proposed model. The study’s findings show that inclusive leadership positively influences employees helping behaviors. Moreover, the psychological factors (i.e., safety and engagement) mediate the relationship between inclusive leadership and employees’ helping behaviors. Theoretical and practical implications for managers, practitioners, and organizations are discussed, while study limitations and directions for future research are also highlighted.

## Introduction

An organization’s growth and success largely rely upon its workforce, who perform teamwork by helping and coordinating to achieve the assigned duties and responsibilities effectively. In this way, preferable work-associated outcomes are generated to achieve organizational objectives by encouraging helping behavior (Carmeli et al., 2010). According to Xia et al. (2019) helping behavior is a voluntary action taken to assist others in the task performance and is considered the key component of an organization’s citizenship behavior as it involves assisting and advising other workfellows in the same work settings, even though it’s not officially obligatory (Organ, 1988). Hence, promoting and encouraging helping behavior in the workplaces could be very advantageous for organizations to enhance overall performance and effectiveness, particularly in today’s competitive world (Karolidis and Vouzas, 2019; Lim and Moon, 2020). It is thus extremely imperative to conceptualize the factors which encourage the workforce to help each others in the workplace (Halbesleben and Wheeler, 2015).

Generally, leadership has been highlighted as a crucial factor in fostering positive individual work outcomes, including employees prosocial behaviors such as helping behaviors. More specifically, inclusive leadership, defined as “leaders who exhibit visibility, accessibility, and availability in their interactions with followers” (Carmeli et al., 2010, p. 250), is considered an important predictor of fostering helping behaviors in the workplace. According to Social learning theory (SLT; Bandura, 1972), individuals learn by focusing their attention on role models and learn appropriate behavior through witnessing what is rewarded and what is punished or which actions attract attention and which do not. Building on the tent of social learning theory, we argue that inclusive leaders’ positive attitude toward employees’ inclusiveness, uniqueness, and appraising their struggle and achievement gives employees more motivation to get involved in helping other behaviors.

Furthermore, the current study intends to unearth the key reasons why inclusive leadership is conducive to promoting employees helping behaviors. We deemed that two psychological mechanisms, i.e., psychological safety and psychological engagement, are more important in translating the impact of inclusive leadership toward employees helping behaviors. First, prior studies (see, for example, Randel et al., 2018; Shore et al., 2018) indicate that in the presence of inclusive leadership, individuals are treated as insiders through values like belongingness and uniqueness. Consequently, inclusive leaders create an environment of working with their employees (Hantula, 2009), where employee input is genuinely valued (Carmeli et al., 2010). Also, inclusive leadership respects employees’ self-value by encouraging them to provide their opinions and views (Carmeli et al., 2010) and hence improves their psychological safety perceptions (Detert and Burris, 2007). Secondly, leaders’ behaviors act not merely as a source of satisfaction and motivation but also nurture a healthy work environment to foster individuals work engagement. Since leadership is viewed as a major factor affecting employees work engagement (Bakker et al., 2011a), we therefore reveals that inclusive leadership helps to enable employees engagement which further promote their prosocial behaviors. In sum, based on SLT, this study not only examine the direct inclusive leadership-helping behaviors relationships, but also unearth two important underlying mechanisms.

**FIGURE 1:**
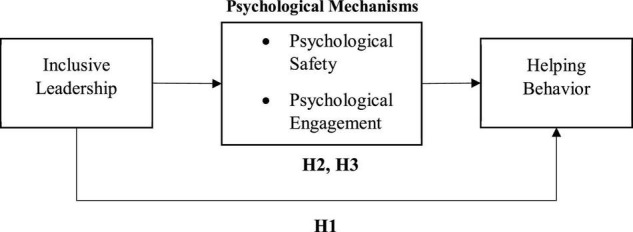
Proposed model.

Taken together, the present study makes the following contributions to organizational behavior and leadership literature. First, this is the first attempt, to our knowledge, that tests the inclusive leadership and employees’ helping behavior relationship. Our results contribute to the literature on both the antecedents of employees’ helping behavior and the outcomes of inclusive leadership. Second, the study explores the underlying mechanisms of psychological engagement and psychological safety in shaping the association between inclusive leadership and helping behavior (see Figure 1). Finally, this study significantly examines the above-mentioned relationships that further clarify how IL is beneficial to helping behavior. Besides conducting this research in Pakistan, it offers unique findings in the non-Western context.

## Theoretical Background and Hypotheses Development

### Inclusive Leadership

Inclusive leadership was defined in terms of the leader’s words and actions in order to encourage and appreciate the subordinates to show their contribution to the achievement of organizational objectives (Guo et al., 2020). The main focus of this leadership is to develop an interactive relationship among leaders and their subordinates by listing and paying increased attention to understanding the employees’ problems and finding an appropriate solution. In this way, a harmonious relationship is promoted that enhances the involvement of subordinates in organizational affairs with an increased level of effectiveness, openness, and availability (Carmeli et al., 2010; Guo et al., 2020).

Inclusive leadership has been identified as a critical element influencing psychological engagement and psychological safety (Blomme et al., 2015). According to studies on the influence of leaders on employee engagement, leaders lead to increased employee engagement in work in a variety of methods (Guo et al., 2020). Leaders, for example, may act as role models for committed activities (Carmeli et al., 2010). Furthermore, leaders may offer assets such as time, money, and knowledge that are required for the engaged effort. Third, leaders may motivate and excite their colleagues to become more engaged (Blomme et al., 2015). Inclusive leadership enables workers to work freely and contribute in decision-making. Leaders appreciate their people, acknowledge their worth, comprehend their requirements, and offer assistance and guidance (Wang and Shi, 2021).

Furthermore, from an equity standpoint, the essence of inclusive leadership is to treat everyone equally in various circumstances (Javed et al., 2019). According to the cultural foundation viewpoint, employees should be receptive to diverse beliefs and actions and forgiving of mistakes (Zeng et al., 2020). Thus, inclusive leadership is a helpful, participatory, equitable, and fault-tolerant leadership approach and a key organizational background factor with a substantial influence on subordinate attitudes (Carmeli et al., 2010).

In the previous literature on business management, inclusive leadership has been paid increased attention by different studies (Qi and Liu, 2017; Qi et al., 2019; Guo et al., 2020; Zhu et al., 2020). These research studies focused on predicting employees’ behavior in the light of inclusive leadership on the basis of different parameters of behavior. Such as Guo et al. (2020) investigated the impact of inclusive leadership on employees’ voice behavior with moderating role of power distance. In the same way, Zhu et al. (2020) investigated that how inclusive leadership may contribute toward employees innovative and creative behaviors in organizations. All these research studies encourage the importance of inclusive leadership for employees’ behavior; however, literature indicates that insights about the impact of inclusive leadership on helping behavior of employees.

### Inclusive Leadership and Employees’ Helping Behaviors

Helping behavior of employees in any organization is defined as a voluntary action performed to assist other coworkers to solve issues related to a specific task (Frazier and Tupper, 2018). For the organizational performance and effectiveness, the helping behavior of employees is considered the main factor in promoting organizational citizenship behavior. In this way, organizational goals are attained more efficiently and sustainably. In the past research, helping behavior has been paid attention to determine its role in the performance effectiveness of the organization (Campbell et al., 2016; Javed et al., 2019).

According to SLT, when leaders provide their assistance to the employees to accomplish different tasks, employees also show their active involvement toward the development of helping behavior to improve organizational functions (Spreitzer et al., 2005; Cangiano et al., 2019). Therefore, the main focus of inclusive leadership is to develop a workplace environment with an increased level of belongingness in which the helping behavior is promoted. The SLT suggests the development of behavioral patterns by learning from the perceptions and actions of others. Therefore, if leadership emphasizes the importance of helping behavior, employees will ultimately intend to follow and learn the positive norms (Nguyen et al., 2019). Accordingly, we hypothesize that:

**H1:** Inclusive leadership positively influence employees’ helping behavior.

### Inclusive Leadership, Psychological Safety, and Employees’ Helping Behavior

The concept of psychological safety indicates the perception of the consequences of interpersonal risks taken in the workplace (Mansoor et al., 2021). It describes a sense of confidence that an individual’s self-image, career, and status will not be negatively influenced if he intends to perform a specific behavior in the relevant work environment. Edmondson (2004) elucidated this concept with a clear distinction between psychological safety and trust. Because the main focus of psychological safety is on self while trust indicates the main focus on others. Hence, in an organization, the employees intend to ensure that they will not be rejected, punished, or embarrassed if they perform a certain behavior by interacting with others. In this way, they make a defensive confirmation with self-safety concerns and ideas. Therefore, increased psychological safety levels motivate the employees to perform a specific behavior more effectively (Yin, 2013).

In the perception of psychological safety, the role of leaders’ behavior is indispensable due to its significant contribution to the employees’ feelings and attitudes. In this regard, inclusive leadership is closely associated with psychological safety due to increased support for subordinates (Burris, 2012; Takeuchi et al., 2012). Inclusive leaders intend to conduct open communication and appreciate their subordinates for good performance, due to which the employees are encouraged and motivated to engage in positive behavior (Zeng et al., 2020). Moreover, employees’ interests, expectations, and feelings are highly valued. Hence, employees perceive that they are safe and secure with less risk of negative outcomes in performing job roles. According to the SLT, the employees not only observe and follow a specific behavior of their leaders but also learn rules of modeled behavior and then use these rules to engage in their preferred behavior (Bandura and Walters, 1977; Chen et al., 2019). In the previous studies (Carmeli et al., 2010; Qi and Liu, 2017; Javed et al., 2019; Mansoor et al., 2021), the impact of inclusive leadership on employees’ behavior with mediating role of psychological safety was determined in the context of voice behavior, innovative work experience, employee involvement, and creative behavior. Based on the aforementioned discussion, we present the following hypothesis:

**H2:** Psychological safety mediates the relationship between inclusive leadership and employee’s helping behaviors.

### Inclusive Leadership, Psychological Engagement, and Employees’ Helping Behavior

Employee engagement is defined as the development of a positive cognitive state of mind to perform the assigned job tasks (Bakker et al., 2011b; Choi et al., 2015). This state of mind is described on the basis of three main dimensions, including dedication, vigor, and absorption. Dedication demonstrates the level of employees’ involvement by recognizing their performance including inspiration and enthusiasm. Vigor illustrates the energy level of the employee, their mental resilience, along with how willing they are to accept challenges and giving in efforts enthusiastically. Lastly, absorption signifies how concentrated and engrossed the employee is in work (Schaufeli and Bakker, 2010; Choi et al., 2015). Positive outcomes pertaining to improved organizational behavior, individual productivity, employee turnover, managerial effectiveness and customer satisfaction are an outcome of a positive relationship between employee and business objectives which is developed as a consequence of employee engagement which is a primary element for business success (Blomme et al., 2015). Accordingly, organizations aim to boost employee engagement in the allocated roles and duties so that the organizational performance is enhanced.

Leadership is a crucial indicator of employees’ engagement as it is directly related to employee motivation and satisfaction level achieved by a supportive work environment. Hence, the establishment of a healthy relationship among the employees is emphasized by inclusive leadership because of its concentration on the requirements, expectations, and interests of subordinates as well as it assures that the resources are available and accessible (Rodriguez, 2018). Based on this, the theory of social exchange states that a reciprocal link between employee and leader exists due to the employees receiving socio-emotional assistance and resources from their leader. A state of positive psychology emerges as they feel obligated to compensate by performing better and giving high productivity. As a result, they also support their coworkers for organizational success (Choi et al., 2015).

Moreover, according to the SLT, positive leader behaviors such as inclusive leadership serve as role models in caring for the well-being of others, which leads them to more engagement at work, as previous studies suggested, and their followers will emulate the leaders’ exemplary behaviors and become prosocial toward their organizations and coworkers (Yaffe and Kark, 2011; Demirtas and Akdogan, 2015). Based on this discussion, we conclude the following:

**H3:** Employee engagement mediates the relationship between inclusive leadership and employees’ helping behaviors.

## Methodology

### Participants and Procedure

The hypotheses of the current study were tested with the data collected through questionnaires from employees (i.e., nurses) working in the health sector of Pakistan. The researchers contacted the human resource department managers of hospitals. They explained the objectives and procedures of the survey, informed their employees, and encouraged them to participate in the study. All the employees were informed that the purpose of the current study is to conduct academic research only, and the data will be kept confidential, and were explained that their participation is voluntary.

The data were collected in three time-lag in a total duration of 2 months for the purpose of avoiding common method bias (CMB) issues (Podsakoff et al., 2003). A unique identifier was assigned to each questionnaire to match the participants responses of time one (T1), time two (T2), and time three (T3). In T1, a questionnaire was consisted of demographic information and the items of the independent variable (inclusive leadership), while in T2, a questionnaire was consisted of mediating variables (psychological safety and psychological engagement) items. Finally, in T3, the questionnaire included items of the dependent variable (helping behaviors). We distributed 715 questionnaires in T1 and got back a response of 520 respondents. In T2, questionnaires were distributed among those who responded in T1, and 485 of them responded. Finally, in T3, the questionnaire was distributed among those who responded in T2 and got back 423 responses. Fourteen questionnaires were not properly filled, which were removed, and the final useable sample was 409. The response rate in the current study was 57.20%. The information regarding participants’ demographics are reported in Table 1.

**TABLE 1:** Participants’ information.

Variables	Categories	Number	Percentage
Gender	Male	179	43.8
	Female	230	56.2
Age (in years)	<24	113	27.6
	24–29	208	50.9
	30–35	73	17.8
	>35	15	3.7
Education	Secondary school or below	23	506
	Bachelor	184	45.0
	Masters	157	38.4
	Others	45	11.0
Work experience (in years)	<1	55	13.6
	1–4	152	37.2
	5–8	105	25.7
	9–12	44	10.8
	>12	53	13.0

### Measures

#### Inclusive Leadership

Inclusive leadership was measured with a nine-item scale adapted from the study of Carmeli et al. (2010). The sample items are “The doctor/boss is open to hearing new ideas” and “The doctor/boss is available for professional questions I would like to consult with him/her.” The Cronbach’s α value in their study was 0.94.

#### Psychological Safety

Psychological safety was measured with a five-item scale adapted from the study of Edmondson (1999). The sample items are “I am able to bring up problems and tough issues,” and “It is easy for me to ask other members of this hospital for help.” The Cronbach’s α value in their study was 0.74.

#### Psychological Engagement

The psychological engagement was measured with nine-item adapted from the study of Schaufeli et al. (2002). The sample items are “When I get up in the morning, I feel like going to work,” and “When I am working, I forget everything else around me.” The Cronbach’s α value in their study was 0.90.

#### Helping Behaviors

The helping behavior variable was assessed with seven-item adapted from Van Dyne and LePine (1998). Two sample items are “I used to help others in their work responsibilities,” and “I help new employees in my hospital.” The Cronbach’s α value in their study was 0.95.

#### Control Variables

The current study considered gender, age, education, and experience as control variables because the previous literature have controlled and shown influence on helping behaviors of employees (Ng and Feldman, 2008; Waismel-Manor et al., 2010). Therefore, we also controlled age, gender, experience, and education in the current study.

### Data Analysis

Structural equation modeling (SEM) was employed in AMOS (version 22.0) to examine the measurement and structural model of the study. SEM is a powerful statistical tool to evaluate the measurement and structural model (Hair et al., 2010), and hence, it is applied to test the proposed hypotheses. Furthermore, recent studies of organizational psychology and behaviors also used SEM to evaluate their study model or hypotheses (Islam et al., 2020; Usman et al., 2021a).

## Results

### Measurement Tests

Statistical remedies were also evaluated in the current study recommended by Podsakoff et al. (2003), to assess the CMB issue. First, Harman’s single factor technique was used, and the results show that the first factor explained 39.21% of the variance of the total variance below the threshold value of 50%, indicating CMB is not an issue. Second, all the variable inter-correlations coefficients were less than 0.90, which further confirmed that CMB is not an issue in the current research.

To examine the reliabilities and validities of the instruments, Cronbach’s alpha (CA), composite reliability (CR), average variance extracted (AVE), and factor loadings were assessed (Fornell and Larcker, 1981; Hu and Bentler, 1999; Hair et al., 2007). In Table 2, CA and CR values are greater than the criterion value of 0.70, indicating that the instruments are reliable. Further, Factor loadings and AVE values are greater than the criterion values of 0.60 and 0.50, respectively, justifying the convergent validity of the instruments. Moreover, all the construct are discriminately valid because the inter-correlations of the study variables are lesser than the value of AVE’s square roots (see Table 3). Hence, these statistics signifies that the study measures are valid and reliable to proceed for further analysis of the proposed relationship.

**TABLE 2:** Measurement model results.

Variables	Factor loadings	Cronbach’s α	Composite reliability	AVE
Inclusive leadership	0.715–0.906	0.955	0.955	0.702
Psychological safety	0.763–0.847	0.900	0.901	0.646
Psychological engagement	0.663–0.794	0.957	0.956	0.564
Helping behaviors	0.727–0.895	0.931	0.930	0.655

**TABLE 3:** Descriptive statistics.

	Mean	SD	1	2	3	4	5	6	7	8
1. Gender	1.56	0.49	1							
2. Age	1.98	0.78	−0.130**	1						
3. Education	2.55	0.76	−0.129**	0.035	1					
4. Exp.	2.73	1.21	−0.004	0.045	0.035	1				
5. IL	4.98	1.56	0.017	−0.022	0.072	0.000	**0.838**			
6. PE	4.95	1.26	−0.010	−0.075	0.007	0.059	0.501**	**0.751**		
7. PS	4.49	1.39	0.088	0.069	−0.010	0.067	0.281**	0.371**	**0.803**	
8. HB	4.83	1.50	−0.063	−0.085	−0.038	−0.022	0.318**	0.391**	0.436**	**0.809**

N = 409, **p < 0.01, IL, Inclusive Leadership; PE, Psychological Engagement; PS, Psychological Safety; HB, Helping Behaviors. The bold values are the square root of AVE.

Moreover, Table 4 reports the results of Heterotrait-Monotrait (HTMT) ratios. According to Henseler et al. (2015), HTMT is an another technique that examines the discriminant validity of the model. The discriminant validity can be established when the ratios of HTMT for each variable is less than 0.85 (Henseler et al., 2015). In this case, all the values are under the suggested threshold, indicating that the discriminant validity is established.

**TABLE 4:** Heterotrait-monotrait ratio (HTMT).

	HB	IL	PE	PS
HB				
IL	0.337			
PE	0.414	0.523		
PS	0.477	0.304	0.400	

*IL, Inclusive Leadership; PE, Psychological Engagement; PS, Psychological Safety; HB, Helping Behaviors.*

### Correlations Results

Table 3 demonstrates the study variables’ means, standard deviations, and inter-correlations. The results show that all the correlation relationships are in the anticipated directions. Table 3 also demonstrates that none of the demographic variables significantly correlate with the study variables.

### Hypotheses Testing

The measurement model fitness indices are assessed by running measurement model in AMOS 22.0. The measurement model results show that the model significantly satisfy model fitness criteria (Hu and Bentler, 1999; Hair et al., 2007), the results are as: (χ^2^/df = 1792.572/655 = 2.737, CFI = 0.916, RMSEA = 0.065, and SRMR = 0.041).

**FIGURE 2:**
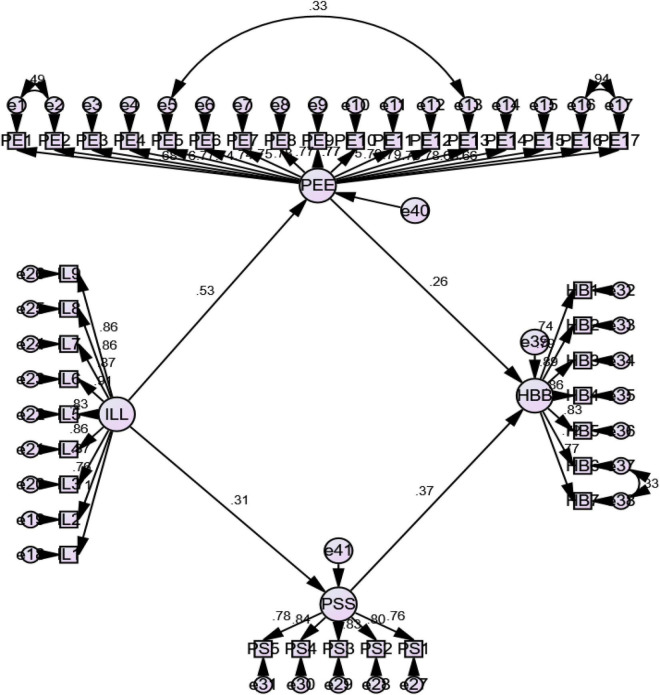
Structural model output.

Next, the study model was converted into a structural model, and first evaluate the model for fitness. The findings of the structural model also indicates that the study model is fit and meet the criteria as (χ^2^/df = 1826.673/656 = 2.785, CFI = 0.914, RMSEA = 0.066, and SRMR = 0.065) (Hu and Bentler, 1999; Hair et al., 2007). Afterward, we then calculated the standardized path coefficients through the application of maximum likelihood procedure in AMOS 22.0 for the suggested structured relationships (see Figure 2). Our study results reveals the significant positive effect of inclusive leadership (β = 0.124, *p* < 0.05) on helping behaviors, henceforth H1 is confirmed. To check the mediation effect (see Table 5), we calculated the bootstrap interval of the indirect effect of inclusive leadership on helping behaviors through psychological safety [β = 0.106, 95% CI (0.073, 0.148)] and psychological engagement [β = 0.155, 95% CI (0.104, 0.215)]. These intervals do not include 0; hence, the mediations were significant, and thus, H2 and H3 were confirmed.

**TABLE 5:** Indirect effects.

Path	Indirect effect	S.E.	UBCI 95%	LBCI 95%	*P*-value	Decision
IL→ PS→ HB	0.106	0.023	0.073	0.148	0.000	Confirmed
IL→ PE→ HB	0.155	0.034	0.104	0.215	0.002	Confirmed

*IL, Inclusive Leadership; PE, Psychological Engagement; PS, Psychological Safety; HB, Helping Behaviors; UBCI, Upper Bond Confidence Interval; LBCI, Lower Bond Confidence Interval.*

## Discussion

Based on social learning theory, the current study hypothesized the influence of inclusive leadership on employee’s helping behaviors. Also, to better understand the mechanism of the association between inclusive leadership and individuals helping behavior, psychological safety and psychological engagement were hypothesized as mechanisms. The study’s finding demonstrates that inclusive leadership positively influences employees’ helping behaviors. Further, the finding also posits that psychological safety and psychological engagement mediate the relationship of inclusive leadership and helping behaviors.

### Theoretical Implications

The current study’s findings contribute on several grounds to Social learning theory, leadership, and helping behaviors literature. Based on the SLT lens, the findings show that inclusive leadership is crucial particularly in exhibiting employee’s prosocial behaviors. SLT reveals that individuals learn by focusing their attention on role models (i.e., teachers, parents, or leaders) and learn appropriate behavior through witnessing what is punished and what is rewarded or which actions attract attention and which do not (Bandura, 1972). Prior studies (i.e., Mayer et al., 2010) have also demonstrated that role models in an organization influence employees’ prosocial (such as helping others) behaviors.

Further, our findings are consistent with the previous studies’ findings that inclusive leadership leads to positive outcomes such as voice behavior, innovative work experience, employee involvement, and creative behavior (Carmeli et al., 2010; Javed et al., 2017; Qi and Liu, 2017; Mansoor et al., 2021). Similarly, the findings in the congruence of other positive leadership styles like participative leadership and helping behaviors (Usman et al., 2021b), the present study findings also reveal that inclusive leadership boosts employees positive workplace behaviors, i.e., helping behaviors. Since inclusive leadership is a critical factor in promoting a harmonious working environment where employees help each other will contribute to high individual and organization level performance.

In addition, the indirect effect of inclusive leadership and helping behaviors through psychological safety and psychological engagement have also been confirmed, which is a further contribution to the helping behavior literature. The findings confirm the process view of leadership by displaying that inclusive leadership shapes the individuals perception (i.e., psychological safety and psychological engagement) about the organization context in a way that encourages helping behaviors. Our approach aligns with some existing studies that advance the notion that leadership influences employees’ workplace behaviors through individual-level factors like intrinsic motivation, creative self-efficacy, and psychological empowerment (Afsar and Umrani, 2019; Javed et al., 2019).

Finally, our findings also expanded the social learning view while explaining the relationship between inclusive leadership and employees helping behaviors. The SLT suggests the development of behavioral patterns by learning from the perceptions and actions of others. Therefore, if leadership emphasizes the importance of openness, participation, and availability, employees will ultimately intend to follow and learn the positive norms (Nguyen et al., 2019). Also, social learning occurs when individuals feel valued in the workplace *via* inclusive leadership attributes such as openness, accessibility, and availability. Employees tend to adopt and replicate such treatment in terms of helping behaviors.

### Practical Implications

The current study have several implications for organizations. First, as inclusive leadership leads to positive work behaviors such as helping behaviors, managers need to understand how to increase these behaviors. Managers should adopt an inclusive leadership style by stressing availability, accessibility, and openness in order to trigger individuals helping behaviors. This is practically essential for leaders to initiate training programs and socialize to nurture a close connection with their employees, as suggested by Javed et al. (2019). Second, the mediating role of psychological safety and engagement highlighted fostering and energizing employees for extra-role behaviors like helping others. Again, managers must focus on an inclusive leadership style, which contributes to developing positive psychological factors and in turn, employees respond in terms of positive work behaviors.

Furthermore, it is important that inclusion is supported at all organizational levels but specifically by top management (Sabharwal, 2014). Inclusive leaders can serve as role models who mentor others in ways that facilitate and promote helping behaviors among organizational members. Also, leaders at all organizational levels can directly reinforce behaviors that support and encourage helping behaviors and which contribute to individual and organizational success.

### Limitations and Future Direction

Drawing conclusions from the results, several issues related to this study need to be noticed. Firstly, although the data were collected in three time-lags, it is difficult to infer cause and effect relationships in this study. Even though a sound theoretical ground is provided for the hypothesized model, future studies should investigate the current model through longitudinal design to allow for stronger causal interpretations of the current study model. Secondly, as we have focused only on the inclusive leadership, psychological engagement, and psychological safety role in explaining employees’ helping behaviors, other unobserved may also be important in explaining helping behaviors, limiting the implications of the current study. Future studies could integrate complementary theories and explanations of helping behaviors of employees. For example, situational factors (relational leadership, authentic leadership) and personal factors (self-efficacy, psychological entitlement), and job characteristics could be explored.

Thirdly, the current study used self-reported measures to assess the study variable, which CMB could accompany. The research is based on self-reported data, and scholars believe it has little validity. However, to lessen such problems, procedural and statistical remedies were followed suggested by Podsakoff et al. (2003). For instance, measuring the predictors and criterion variable in different time-lags, ensuring anonymity and confidentiality, Harmans’ single factor test, and confirmatory factor analysis exhibited no issue of CMB. However, we admit that one cannot completely determine the magnitude, and hence, a longitudinal study and collecting data from different sources are desirable.

## Conclusion

The current study develops the understanding of a relatively under-researched form of leadership (i.e., inclusive leadership) and its potential role in explaining employees’ helping behaviors. Further, the present study also examined the mechanism (i.e., psychological safety and psychological engagement) through which inclusive leadership influences employees’ helping behaviors. The findings show that inclusive leadership enhances psychological safety and psychological engagement and, in turn, increases employees’ helping behaviors. Finally, based on the limitations of the study, future scholars may work to fill the existing gaps in the current literature.

## Data Availability Statement

The raw data supporting the conclusions of this article will be made available by the authors, without undue reservation.

## Author Contributions

SQ took the overall responsibilities of the manuscript and gave the idea of the issue to be investigated. MU wrote the introduction part. UG worked on literature review section of the manuscript, took the responsibility of data collection, helped in the methodology part, and ran the statistical analysis. KK compiled the discussion part and provided a technical support throughout the manuscript. All authors contributed to the article and approved the submitted version.

## Conflict of Interest

The authors declare that the research was conducted in the absence of any commercial or financial relationships that could be construed as a potential conflict of interest.

## Publisher’s Note

All claims expressed in this article are solely those of the authors and do not necessarily represent those of their affiliated organizations, or those of the publisher, the editors and the reviewers. Any product that may be evaluated in this article, or claim that may be made by its manufacturer, is not guaranteed or endorsed by the publisher.
